# Integrated Control and Management of Neglected Tropical Skin Diseases

**DOI:** 10.1371/journal.pntd.0005136

**Published:** 2017-01-19

**Authors:** Oriol Mitjà, Michael Marks, Laia Bertran, Karsor Kollie, Daniel Argaw, Ahmed H. Fahal, Christopher Fitzpatrick, L. Claire Fuller, Bernardo Garcia Izquierdo, Roderick Hay, Norihisa Ishii, Christian Johnson, Jeffrey V. Lazarus, Anthony Meka, Michele Murdoch, Sally-Ann Ohene, Pam Small, Andrew Steer, Earnest N. Tabah, Alexandre Tiendrebeogo, Lance Waller, Rie Yotsu, Stephen L. Walker, Kingsley Asiedu

**Affiliations:** 1 Skin NTDs Program, Barcelona Institute for Global Health, Hospital Clinic-University of Barcelona, Barcelona, Spain; 2 Division of Public Health, School of Medicine and Health Sciences, University of Papua New Guinea, Port Moresby, Papua New Guinea; 3 Clinical Research Department, Faculty of Infectious and Tropical Diseases, London School of Hygiene & Tropical Medicine, London, United Kingdom; 4 Hospital for Tropical Diseases, University College London Hospitals NHS Trust, London, United Kingdom; 5 Neglected Tropical and Non Communicable Diseases Program, Ministry of Health, Government of Liberia, Liberia; 6 Department of Control of Neglected Tropical Diseases, World Health Organization, Geneva, Switzerland; 7 The Mycetoma Research Centre, University of Khartoum, Khartoum, Sudan; 8 International Foundation for Dermatology, London, United Kingdom; 9 Anesvad foundation, Bilbao, Spain; 10 Leprosy Research Center, National Institute of Infectious Diseases, Tokyo, Japan; 11 Fondation Raoul Follereau, Cotonou, République du Bénin; 12 Medical Department, German Leprosy and TB Relief Association, Enugu, Nigeria; 13 Department of Dermatology, Watford General Hospital, Watford, United Kingdom; 14 World Health Organization Country Office, Accra, Ghana; 15 Department of Microbiology, University of Tennessee, Knoxville, Tennessee, United States of America; 16 Group A Streptococcal Research Group, Murdoch Children’s Research Institute, Melbourne, Victoria, Australia; 17 National Yaws, Leishmaniasis, Leprosy and Buruli ulcer Control Programme, Ministry of Public Health, Yaoundé, Cameroon; 18 World Health Organization Regional Office for Africa, Brazzaville, Congo; 19 Department of Biostatistics and Bioinformatics, Rollins School of Public Health, Emory University, Atlanta, Georgia, United States of America; 20 Department of Dermatology, National Center for Global Health and Medicine, Tokyo, Japan; Swiss Tropical and Public Health Institute, SWITZERLAND

## Introduction

Neglected tropical diseases (NTDs) are communicable diseases that occur under conditions of poverty and are concentrated almost exclusively in impoverished populations in the developing world. NTDs affect more than 1000 million people in tropical and subtropical countries, costing developing economies billions of dollars every year. Effective control of NTDs can be achieved with the use of large-scale delivery of single-dose preventive chemotherapy (PC) or intensified disease management (IDM) or both, as is the case for some diseases such as lymphatic filariasis, trachoma, and yaws.

Several NTDs exhibit significant cutaneous manifestations that are associated with long-term disfigurement and disability, including Buruli ulcer (BU); cutaneous leishmaniasis (CL); leprosy; mycetoma; yaws; hydrocele and lymphoedema (resulting from lymphatic filariasis); and depigmentation, subcutaneous nodules, severe itching, and hanging groin (resulting from onchocerciasis). Skin examination offers an opportunity to screen people in the communities or children in schools to identify multiple conditions in a single visit. This common approach to skin diseases justifies the integrated delivery of health care interventions to both increase cost-effectiveness and expand coverage.

WHO’s Department of Control of NTDs (WHO/NTD) plans to promote an integrated strategy for the skin NTDs requiring IDM. Targeting skin NTDs also provides a platform for treatment of common skin conditions and, therefore, has wider public health benefits. An informal panel of experts (writing this manuscript) was established to help develop guidance in support of the new WHO strategic direction and to develop a proposal for a change in policy for the integrated control and management of the skin NTDs.

A symposium at the 2015 ASTMH meeting[1] initiated a discussion of opportunities around integration of surveillance and control of NTDs that affect the skin, but this paper moves these ideas forward and includes some initial recommendations about how these opportunities could be realised. We aim to provide specific pragmatic information and actual recommendations about potential surveillance and management approaches.

## Burden of Skin NTDs

Skin NTDs are frequently co-endemic in many countries, districts, and communities (Table 1). [2–9] While none of the skin NTDs are significant causes of mortality, they are responsible for a large number of disability-adjusted life years (DALYs) lost.[10] For example, contractures and resulting disability in BU, advanced lymphoedema and hydrocele in LF, the consequences of permanent nerve damage in leprosy, amputations in mycetoma, and bone involvement in yaws can lead to debilitating deformities and difficulty in securing employment.[11]

**Table 1:** Characteristics of skin NTDs.

	Causative agent	Mode of transmission	Natural reservoir	Geographic distribution by continent/region (Major affected countries)	Key manifestation	Complications	Peak age (male: female ratio)	Incidence (annual) year ^6–13^	WHO target by 2020	WHA resolution
**Buruli ulcer**	*Mycobacterium ulcerans*	Unknown	Contaminated water	West and Central Africa, Western Pacific	Skin ulcer	Severe scarring with limb contractures	5–15 (2:1)	2,200	Control	WHA57.1 (2004)
**Cutaneous leishmaniasis**	*Leishmania* spp.	Sand fly vectors	Rodents, Hyraxes	Middle East, West and East Africa, Mediterranean basin, and South-America	Skin ulcer, papules, nodules or plaques,	Disseminated skin disease and significant facial destruction	All ages (1:1)	700,000	Control	WHA60.13 (2007)
**Filarial lymphoedema**	Filariae such as *Wuchereria bancrofti*	Anopheles, Culex and Aedes mosquitoes	Human	Worldwide distribution	Lower limb oedema	Lymphoedema and elephantiasis	Adults (ND)	970,000	Elimination as public health problem	WHA50.29 (1997)
**Onchocerciasis complications**	*Onchocerca volvulus*	Blackfly Simulium vectors	Human	West, Central and East Africa, foci in Latin America	Itchy papules, vesicles, pustules, papulonodules or plaques Subcutaneous nodules; hanging groin	Impetigo Physical appearance, nuisance, psychological impact, stigma	Children and adults (ND) Adults (ND)	NA	Elimination in selected countries in Africa	NA
**Leprosy**	*Mycobacterium leprae Mycobacterium lepromatosis*	Probably respiratory route	Human	Worldwide distribution (India, Brazil, Bangladesh, Indonesia, DRC, Ethiopia and Nigeria)	Skin patches/nodules, Thickened nerves, Sensory and/or motor disturbance	Peripheral neuropathy and permanent damage of the limbs, eyes and nose	5–15 and 20–40 (1.5:1)	215,000	Elimination as public health problem	WHA51.15 (1998)
**Mycetoma**	Fungal or bacterial species	Inoculation via contaminated thorn or splinter	Soil	Worldwide distribution (Sudan, Mexico and India)	Subcutaneous mass with sinuses and discharge	Local destruction of subcutaneous tissue	All ages (3:1 to 5:1)	Unknown	Control	WHA69.21 (2016)
**Yaws**	*Treponema pallidum* ssp. *pertenue*	Direct contact	Human	West and Central Africa and South Pacific	Skin ulcer	Involvement of the bones and joints	2–15 (1.5:1)	60,000	Eradication	WHA31.58 (1978)

In addition, skin NTDs result in stigmatization, discrimination, and psychological distress, which contribute to suffering and may affect health-seeking behaviours and adherence to treatment.[12] Finally, the economic impact of accessing care and rehabilitative measures can be substantial.[13]

## Policy Change

In May 2013, the World Health Assembly (WHA) adopted resolution WHA66.12, which calls on Member States to intensify and integrate control measures to improve the health of NTD-affected populations.[14] Individual NTDs have WHA mandates, including the control of morbidity due to BU, CL, filarial lymphoedema, the elimination of onchocerciasis, the achievement of elimination of leprosy as a public health problem, and the eradication of yaws. In May 2016, the WHA adopted a resolution on mycetoma that called for the need to develop diagnostic tests and simpler treatment as well as enhanced surveillance.[15]

For many years, vertical disease programmes were established to deal with priority diseases, but, increasingly, there has been a move to integrate programmes into general health services. WHO’s Department of Control of NTDs currently promotes intervention-based approaches rather than disease-specific approaches. Each vertical disease program is resource intensive, and resources are not maximized when they are fragmented. Integrating interventions should allow a common approach for case detection and community-based diagnosis, resulting in increased program efficiency through sharing of resources. We propose a new approach to neglected tropical skin diseases, in which seven diseases are grouped together. Integration is defined here to mean combining activities of two or more diseases at the same time and in the same communities with the aim of increasing efficiency. Each country and region may adapt the strategy to the prevailing local or regional co-endemicity of these diseases.

The following are reasons why a policy change to the integrated approach for skin NTDs is feasible.

- Skin examination is an opportunity to identify multiple conditions in a single visit.
- Skin diseases can be suspected and diagnosed clinically by appropriately trained individuals, including community health workers and village volunteers.
- The case-management strategy of the skin diseases targeted is similar, including detection and diagnosis by skin examination, with or without confirmation of the diagnosis by laboratory test, and treatment by the use of effective medicines (oral treatment and/or injection) or morbidity and disability management.

## Benefits and Challenges

The proposed integrated strategy may provide many benefits and opportunities:

1. Increased effectiveness and efficiency.
2. Increased impact of resources improving the opportunity and justification for investment.
3. Increased access to timely diagnosis of cases from the communities thus enhancing disease surveillance.
4. Alleviation of poverty as a result of morbidity caused by NTDs.
5. Improved knowledge, capacity, and motivation of health workers and village volunteers who may see only a few or none of these diseases in single vertical programmes.
6. Sustained awareness and knowledge of both declining and emerging diseases to enhance surveillance.
7. Development of regional centres of excellence.
8. Improvement in skin health overall.

Despite the potential benefits, the following potential challenges should be acknowledged:

1. Loss of vertical programmes may lead to loss of specialized expertise.
2. Lack of adequately trained staff.
3. Staff attrition after training.
4. Referral centres may be unable to cope with the increased demand for skin NTD services.
5. Risk of developing a new vertical programme, which remains poorly integrated with the existing health care system.

## Description of Integrated IDM NTD Implementation

We propose three main linked activities in support of this integrated strategy (Box 1): firstly, identification of areas of geographic overlap; secondly, the use of training packages for the identification of multiple skin conditions; finally, integrated active case detection and use of pathways for diagnosis and management in the local community as far as possible, with referral to local health centres and district hospitals as required.

**Box 1. Integrated IDM-NTDs Implementation Approach**

- **Initial assessment of disease burden:** conduct surveys to identify endemic areas for targeting intensified interventions.
- **Training:** validate a training program based on standardised clinical diagnostic schemes and organise training for trainers, health workers, and village volunteers.
- **Development of an integrated control strategy for each district:** suggest interventions to meet the specific needs of each district, depending on which diseases are identified in the initial assessment and survey.
  - **Social mobilisation:** create demand for and a means of participating in interventions, and address specific aspects and concerns related to the diseases.
  - **Active case detection:** implement active case finding in schools and communities.
  - **Case management:** establish a referral pathway to undertake early diagnosis and treatment.
  - **Health facility mapping and strengthening:** mapping health facilities in endemic areas to guide the needed improvements in infrastructure, equipment, and supplies to ensure optimum quality care of patients.

### Assessment of disease burden

The first step of the integrated approach is to establish the presence or absence of disease in each district for the purpose of deciding the specific intervention(s) that might be required. Initial mapping could be based on a combination of routine surveillance data and specific population-based surveys. These data can be used to classify the Implementation Unit (IU) as a whole as being endemic or nonendemic. Usually the district level is identified as the IU, covering a population of 100,000–250,000, but the choice should be guided by feedback received from lower administrative levels (i.e., if the skin NTD is very focal, a lower administrative level such as sub-district may be chosen as the IU).

Passive surveillance data in health care facilities normally includes the patient’s village of residence, which constitutes the basic mapping unit and allows identification of IUs with current or historical cases of the skin NTDs.[16] However, hidden or unknown cases would not be identified through this approach. Counts from sub-IU regions or point locations of cases during active case-finding can be collected by mobile teams visiting villages in affected areas. Rapid assessment procedures are also emerging as useful tools that provide estimates of the probability of local prevalence (e.g., prob[prevalence > 0.1]) for each IU rather than estimates of the local prevalence itself.[17]

### Training of health workers and village volunteers

The success of an integrated approach will rely on well trained health workers and village volunteers being able to correctly identify multiple skin conditions. It is, therefore, necessary to develop, validate, and implement structured training programmes for those who will be conducting the field work as well as for the staff who will be training them. Simplified algorithms (Table 2) have shown reasonable sensitivity and specificity in diagnosing a limited range of skin conditions when compared to diagnoses made by dermatologists[18,19], but further work is needed to expand these algorithms to cover the full range of common skin conditions and skin NTDs. Simple integrated pictorial guides can also be developed to help health workers and village volunteers. Structured teledermatology resources could provide a system of support.[20] Data collection could be augmented through the use of electronic data collection tools and cloud-based data management, which have proven powerful in large-scale mapping projects.[21]

**Table 2:** An example of key diagnostic signs for identification of targeted diseases.

Key sign identified by HCW or village volunteer	Diagnostic criteria utilised by HCW or referral centre	Common differential diagnosis
**Skin ulcer**	Presence of ulcerative lesions with or without crusts	Buruli ulcer, Cutaneous leishmaniasis, Yaws, Tropical ulcer, Stasis or venous ulcer
	Presence of chronic nodules or papillomatous lesions associated with ulceration	
	Edges raised or indurated in CL and yaws; edges undermined in BU	
**Subcutaneous mass**	Indurated painless swelling or mass involving the foot	Mycetoma, Chromoblastomycosis, Buruli ulcer nodule or plaque, Skin cancer, Kaposi’s sarcoma, Onchocercal nodule
	History of penetrating injury at the same site or walking barefoot in mycetoma	
	Sinus tracts, chronic discharge and grains in mycetoma	
	Well-demarcated firm subcutaneous nodule(s) overlying a bony prominence (e.g., iliac crest, trochanters, ribs, sacrum) in onchocerciasis	
**Swelling of limb or legs**	Painless non-pitting swelling	Filarial lymphoedema, Podoconiosis, TB lymphadenitis, Leprosy oedema, Buruli ulcer oedema, Congestive heart failure oedema
**Skin patch**	Presence of a hypopigmented patch	Leprosy, Pityriasis versicolor, Pityriasis alba, Vitiligo
	Reduced sensation within the patch in leprosy	
	Enlarged nerves in leprosy	
	Chronic duration (>3 months)	

HCW, health care worker.

### Conduct active case detection

Scale-up of case detection activities is critical to effectively reduce the burden and transmission of skin NTDs. Even in NTDs where mass drug administration (MDA) is the initial stage of control interventions—such as yaws—as disease prevalence comes down, incident disease will still occur, for which individual diagnosis and treatment will be required to prevent resurgence.[22]

Different approaches to active case detection may be used. House-to-house screening strategies yield the highest number of newly detected cases, though this strategy can be expensive and difficult to sustain. Alternative strategies include mobile teams visiting villages to screen all attendees at a central location or the use of an incentive-based approach, in which case detection is done by trained health workers detecting cases in their health centre catchment areas. In sub-Saharan Africa, trained village volunteers have also been instrumental in the detection and referral of diseases such as Buruli ulcer, Guinea worm, and leprosy.[23,24] A large network of village volunteers has also been pivotal in the Indian yaws elimination program and the program to eliminate visceral leishmaniasis.[25]

Social mobilization activities will be needed prior to the start of active case detection programs. Communication efforts will focus on informing and enhancing knowledge among the general public to engage people and strengthen their participation in case finding activities. Other social mobilization avenues, including mass media, will provide a common platform by which to address social aspects associated with these diseases such as stigma and discrimination.

### Referral pathways

It will be important to establish clear referral pathways for people with positive findings on screening for both suspected targeted diseases and non-targeted conditions, some of which can be managed at frontline health care level.

Cases of skin NTDs will most often be detected at the community level by health workers or volunteers and then referred to the nearest health facility for management. Cases that cannot be managed at the primary health facility will be referred to the peripheral hospital. At this level, diagnosis and management of minor complications like skin grafting should be done. Complex cases should be referred to specialist referral centres. Community-based rehabilitation programs will need to be strengthened to support the increased case load. Improvements in the public health system are required to make treatment available and accessible at all levels.

### Clinical diagnosis and laboratory confirmation

**Fig 1:**
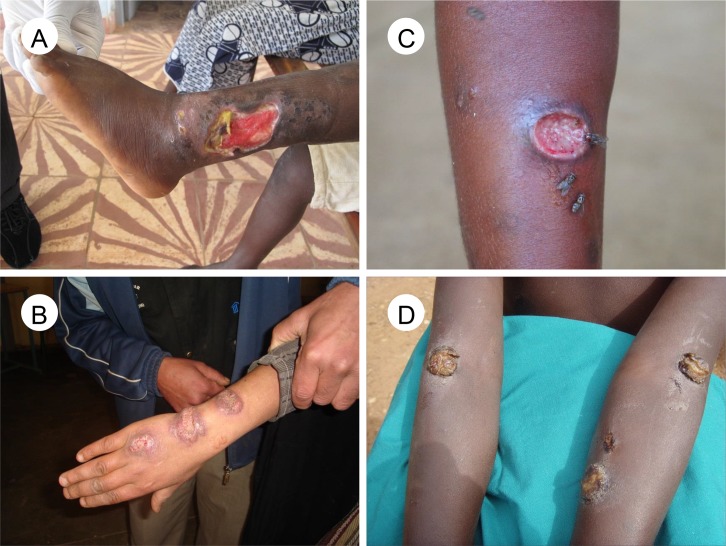
Common skin ulcerative lesions related to neglected tropical diseases. (A) Buruli ulcer with undermined hanging edge, (B) Ill-defined ulcerated infiltrated granulomatous-looking lesions on dorsum of the hand in cutaneous leishmaniasis, (C) Early-stage yaws ulcer with raised edge and “raspberry” type appearance of the central granulation tissue, (D) Multiple yellow-crusted ulcers on the arms in secondary yaws. Images credit: Kingsley Asiedu (A,D), Oriol Mitjà (C), Jorge Postigo (B).

**Fig 2:**
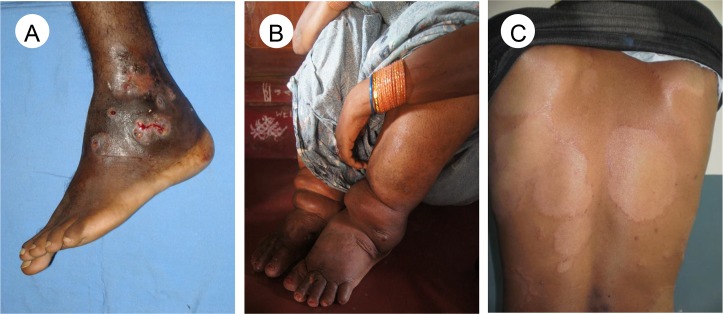
Common skin neglected tropical diseases lesions. (A) Mycetoma with few active sinuses, grains, and discharge, (B) Bilateral lymphoedema of both legs in the late stage of lymphatic filariasis, (C) Hypopigmented anaesthetic macules with infiltrated edge of borderline tuberculoid leprosy. Images credit: Ahmed Fahal (A), CDC Public Health Image library (B), Rie Yotsu (C).

Clinical signs are of variable sensitivity in these diseases, so well-trained staff and diagnostic tests have an important role on diagnosis. The manifestations of leprosy have overlapping clinical features with many other skin diseases.[4] Chronic skin ulcers that fail to heal are a common presentation for all three: BU, CL, and yaws (Fig 1).[2,3,6] Skin ulcers may also result from polymicrobial infections, *Haemophilus ducreyi*,[13] and neuropathy (due to leprosy, diabetes) or vascular disease. Lower limb swelling of filarial lymphoedema may be mistaken for podoconiosis, TB lymphadenitis, or systemic diseases such as heart failure (Fig 2).[7] The main differential diagnoses of mycetoma are chromoblastomycosis and skin cancer.[5]

#### Skin ulcer

The diagnosis of skin ulcers in the tropics remains problematic as clinical features alone are insufficient to make a decision on treatment. PCR diagnostic platforms in reference laboratories are used for confirmation of many conditions, but these facilities are remote from the communities where the diseases occur. Sampling procedures like swabbing for detection of *Mycobacterium ulcerans*,[26] and *Treponema pallidum* subsp. *pertenue*[27] can be performed in the field. Routine diagnosis of CL is based on detection of *Leishmania* spp. DNA in the biopsy of skin lesions;[28] however, it is also possible to perform DNA analysis on impression smears from ulcerated CL lesions that can be collected in the field.[29] The main disadvantage of PCR is that sample transfer mechanisms from the field to reference laboratories for testing are generally slow, resulting in delays and dropout during the diagnostic process.

Point-of-care tests (POCT) are available to aid clinicians to determine the etiology of skin ulcers before the patient leaves the clinical setting. Fluorescent thin layer chromatography (fTLC) is a simple and low-cost technique that can be used for detection of mycolactone in skin swabs from BU lesions at a peripheral hospital laboratory using a small bench analyser;[30] however, this test is still in the development stage. The Dual Path Platform (DPP) yaws rapid test kit, which is based on simultaneous detection of antibodies to treponemal and nontreponemal antigens, allows for serological diagnosis of yaws in the field.[31,32]

#### Subcutaneous mass

Multiple diagnostic tools are usually required to determine the extent of infections and to identify the causative agents of mycetoma and guide treatment. Ultrasound examination, fine-needle aspiration and deep-seated surgical biopsy need to be performed if feasible. The ultrasound and examination of aspirated material can be POCTs. Surgical biopsies can be processed for tissue histopathological examination, microbiology, and molecular studies. Individuals suspected of having mycetoma will need to be referred for further imaging to determine the extent of disease.

Formal diagnosis of onchocerciasis is by skin snips to detect *Onchocerca volvulus* microfilariae. Ultrasound of suspected onchocercal nodules may reveal dead or live adult worms.

#### Limb swelling

Filarial lymphoedema is clinically difficult to distinguish from podoconiosis, but a diagnostic algorithm exists. Clinical diagnosis is accurate in settings where only podoconiosis is endemic; in settings where the two diseases may overlap, the combination of clinical history, physical examination, and blood tests for antifilarial antibody (Wb123 assay) have been used to reach a diagnosis.[33]

#### Skin patch

The diagnosis of leprosy is usually made clinically, which requires health workers to be trained to recognise the varied presentations of the disease including the immune-mediated leprosy reactional states. Skin biopsy is not routinely performed and needs to be interpreted in conjunction with the clinical features. In two leprosy referral centres in Brazil, slit skin smears were only positive in 59%[34] of patients and have not been a recommended part of leprosy programmes since 1998. Patients with suspected leprosy will need to be referred for further assessment and diagnostic procedures where necessary. Individuals suspected of having leprosy need to be assessed for nerve function impairment, and this needs to be repeated regularly during treatment and beyond.

### Treatment

If skin NTDs are diagnosed and treated early, disabilities and disfigurements are preventable. In addition, simple skin-directed therapy can contribute to enhanced resolution and reduction in morbidity.

#### Specific interventions

Once a presumptive diagnosis is established, patients need to be referred for confirmatory diagnosis or testing and treatment except for yaws, which can be immediately treated at the time of detection using single-dose oral drugs (Table 3). Nonopioid analgesics are usually sufficient for managing mild pain related to skin lesions; however, more severe pain may complicate some diseases (e.g., neuropathic pain in leprosy or pain related to erythema nodosum leprosum). For yaws, treatment of all household contacts is necessary, even if they have no symptoms. The treatment of contacts of leprosy patients remains controversial and raises ethical issues around disclosure of diagnosis.

**Table 3:** Recommended diagnosis and management of suspected skin lesions.

	Field assessment	Initial management	Laboratory tools	Medical treatment	Supportive measures	Treatment of contacts	Surgery	Prevention of disabilities and rehabilitation
**Buruli ulcer**	Clinical	Swabbing, registration and referral	PCR of skin swab samples fTLC under development	Oral rifampin + injectable streptomycin or oral clarithromycin for 8 weeks	Wound dressing	No	Yes	Yes
**Cutaneous leishmaniasis**	Clinical	Swabbing, registration and referral	Microscopy and PCR of skin swab	Depends on species. Local or systemic therapy.	Wound dressing	No	No	No
**Filarial lymphoedema**	Clinical	Registration and referral	ICT antigen test (usually negative), and antifilarial antibodies	Oral diethylcarbamazine for 12 days ± doxycycline for 4 to 6 weeks	Skin barrier function improvement measures	No	Yes	Yes
**Oncocerciasis**	Clinical	Registration and referral	Skin snips. Serological and antigen tests under development	Oral ivermectin	Pruritic rash -treatment for any itching and secondary infection	If in endemic area	Yes for nodules or hanging groin	No
**Leprosy**	Clinical	Registration and referral	Slit skin smear or skin biopsy material	Multidrug antibiotic therapy for 6 or 12 months.	Home-based self-inspections and appropriate footwear	Single dose rifampicin is being piloted but is not policy	Yes	Yes
**Mycetoma**	Clinical	Registration and referral	Microscopy examination and culture of grains/biopsy	Depends on species. Long term antibiotic or antifungal.	Skin barrier function improvement measures	No	Yes	Yes
**Yaws**	Clinical	Swabbing, and immediate treatment	DPP test and PCR of skin swab	Single oral dose of azithromycin (2^nd^ line: injectable benzathine penicillin)	Wound dressing	Single dose azithromycin	No	No

fTLC, fluorescent thin layer chromatography; ICT, immunochromatography, DPP, dual path platform yaws assay.

Surgery is only occasionally needed for these diseases. In BU, antibiotic therapy (oral or injectable) is largely replacing surgical excision of tissue in active disease; surgery followed by physical therapy may be required for preventing contractures. In leprosy, surgery has long been used to correct functional and stigmatising cosmetic impairments. Early localised mycetoma lesions are amenable to surgical cure with a lower recurrence rate. Problematic onchocercal nodules can be excised, and hanging groin in onchoceriasis is amenable to surgery to reduce psychological distress.

#### Clinical wound care and repair of skin barrier function

Importantly, integrated but nonspecific interventions can be implemented for case morbidity management that can benefit patients with skin NTDs sharing similar basic pathologies.

Wound management is a common approach for most skin NTDs; hence, the provision of appropriate dressings and training of health workers is important for a satisfactory outcome. Effective wound management requires access to water and simple, cheap non-adherent dressings, which keep wounds clean, protected from trauma, improve healing rates of damaged skin, and potentially prevent transmission.

Skin barrier function improvement measures (e.g., washing, emollients, and compression shoes) minimize the risk of further damage in filarial lymphoedema.[35,36] Provision of simple exercise regimens with or without compression can also improve lymphoedema. The use of shoes is beneficial in the fight against several skin NTDs, and there are likely to be additional benefits such as protection against tetanus, tungiasis, and soil-transmitted helminths.

### Future Directions

An integrated approach to the skin NTDs has the potential to reduce transmission, delays in diagnosis, and associated morbidity of these conditions and promote skin health for all. An integrated approach also has the potential to reduce costs for both patients and health systems. The WHO Department of Control of NTDs should take the lead in coordinating global efforts with the support of donors and partners, focusing on key areas (Box 2). Publication of this policy paper aims to trigger public debate about the approach and to encourage new funding to be targeted towards management of these important NTDs.

**Box 2. Next Steps**

**Advocacy**

- Increasing awareness of skin NTDs and their impact on affected communities.
- Promoting integrated management schemes and their potential benefits to society and donors.
- Networking technical and professional groups, donors, NGOs, endemic countries, and different disease control programmes.

**Policy**

- Gaining consensus and support from all major stakeholders including the Ministries of Health on the way forward for implementation.
- Promoting a common management strategy of these diseases at community and health facility levels and resources required at each level.

**Research**

- Validating a clinical algorithm for identification of skin NTDs using key symptoms and signs.
- Developing common clinical and laboratory diagnostic platforms for these diseases, which are practical in the field.
- Mapping to identify their overlap to allow integrated coordinated control and treatment activities as well as health system strengthening for service delivery.
- Piloting the integrated approach in one or several regions.
- Better understanding of the epidemiology of these diseases including transmission and interaction with poverty and water, sanitation, and hygiene (WASH).
- Understand community resilience and program factors that strengthen community participation.

Integration of surveillance and interventions will not be possible without considerable political support, at a number of levels. There will be very real challenges to integration, including relationships with donors, potential changes to NTD management structures, and complexities in health care worker training among many others, and any of these challenges could derail efforts to achieving integrated management. Strong relationships will be required between governments, international agencies, implementing partners, and donors, with a clear plan of action supported by an evidence base to move forward an agenda of integration.
